# Acanthosis Nigricans Associated with Morbid Obesity and Colorectal Adenocarcinoma

**DOI:** 10.1155/2012/545247

**Published:** 2012-03-26

**Authors:** J. Li, A. Yazdabadi, R. Sinclair

**Affiliations:** Department of Dermatology, St. Vincent's Hospital Melbourne, Fitzroy, VIC 3065, Australia

## Abstract

We report a case of a morbidly obese 32-year-old man presenting with acanthosis nigricans in the setting of Duke's B adenocarcinoma of the hepatic flexure.

## 1. Introduction

Acanthosis nigricans (AN) is characterized by symmetric, hyperpigmented, velvety plaques occurring in the skin flexures or mucosal surfaces. It is the most common dermatological manifestation of obesity and insulin resistance [1]. Other causes are drugs, benign inherited AN, and, most importantly, malignant or paraneoplastic AN [2]. We describe a case of a young man presenting with florid acanthosis nigricans with numerous overlying fibroepithelial polyps in the setting of the comorbidities of colorectal adenocarcinoma and morbid obesity, highlighting the need to consider coexisting underlying malignancy even in cases associated with obesity.

## 2. Case Presentation

**Figure 1:**
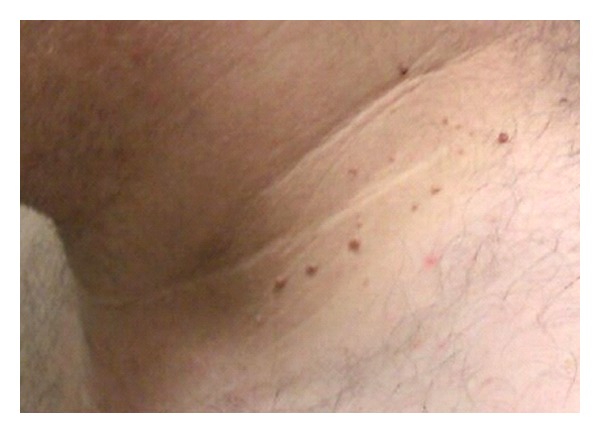
Lateral view of the neck.

**Figure 2:**
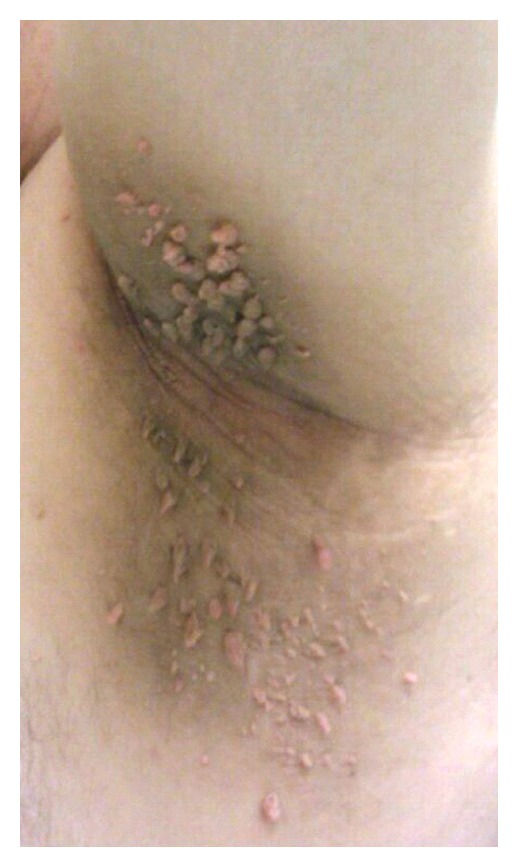
Anterior view of the left axilla.

A 32-year-old man presented with a two-year history of increasing pigmentation in the neck, axillae, and groin with increasing numbers of overlying fibroepithelial polyps. On examination, symmetrical hyperpigmented velvety plaques were present over the neck, axillae, and groin with over 100 fibroepithelial polyps (Figures 1 and 2). Biopsy showed multiple fibroepithelial polyps lined by hyperplastic epidermis with a fibroblastic stroma, and no evidence of malignancy. His palms were examined and found to be normal.

The patient had a history of morbid obesity with a body mass index of 36.4 complicated by severe obstructive sleep apnoea. He had no history of diabetes mellitus. Two years prior to his current presentation, he had been diagnosed with colorectal cancer after he presented with abdominal distension, diarrhea, and haematochezia. Colonoscopy revealed a 3.5 cm mass at the hepatic flexure, and he underwent a right hemicolectomy. Histopathology of the resected specimen showed a Duke's B moderately differentiated adenocarcinoma (T3N0M0). Adjuvant therapy was not recommended. The patient is being followed up with surveillance colonoscopies.

The patient's acanthosis nigricans had persisted after his operation and was not specifically treated. His fibroepithelial polyps were treated with serial snip excisions, and he was referred to his general practitioner for assessment of insulin resistance and institution of weight loss measures.

## 3. Discussion

The association between acanthosis nigricans (AN) and malignancy was first noted in 1890 by two independent observers, Pollitzer and Janovsky [2]. Malignant AN represents a benign dermatosis associated with underlying cancer, gastrointestinal adenocarcinoma in 80% of cases. It is associated less commonly with other adenocarcinomas and rarely with nonepithelial tumours such as sarcomas or lymphomas [3]. Clinically malignant AN may present together with other cutaneous markers of internal malignancy including palmoplantar keratoderma (tripe palms), mucosal papillomatosis, and the sign of Leser Trelat [4]. Development of skin lesions may correlate with progression of malignancy, although they may also precede or follow the underlying diagnosis [5]. Rarely lesions may regress after curative treatment.

Insulin resistance is key to the aetiology of all forms of AN; however, the exact molecular mechanisms remain unclear. In the case of malignant AN, multiple growth factors including transforming growth factor *α*, insulin-like growth factor, and fibroblast growth factor have been implicated. These factors most likely act by exerting an insulin-like effect on keratinocytes and dermal fibroblasts [2].

We report a case of AN associated with both obesity and gastrointestinal malignancy. This case is unusual in a number of respects.

Malignant AN typically develops rapidly in nonobese patients aged over 40 years. It is often associated with tripe palms and other markers of internal malignancy, which were not present in this case [6]. The tumour is usually highly advanced and fatal. Furthermore malignant AN is rarely caused by colorectal carcinoma. In fact it is more commonly associated with adenocarcinoma of the pancreas, biliary tract, and uterus despite the lower prevalence of these diseases [7].

Conversely, AN and skin tags are both common cutaneous dermatoses associated with obesity-related insulin resistance [8]. Obesity is also a risk factor for colorectal cancer, particularly in males, conferring a relative risk of about 1.4 [9]. Thus despite the chronological link between the onset of AN and the diagnosis of cancer, it is difficult to conclude if this patient truly had malignant AN, or obesity-associated AN with coincidental colorectal cancer, especially as there is no difference in the histopathological findings between the two.

Treatment of AN is difficult but should be considered if lesions are disfiguring or symptomatic with pain or pruritus. Retinoids, cyproheptadine, and chemotherapy have been reported to be beneficial [10]. In the setting of obesity, weight loss with appropriate dietary and lifestyle modifications should be encouraged [2].

This case highlights the importance of a systematic history when confronted with patients presenting with AN. Although this case may indeed represent obesity-related AN, it is a reminder that comorbidities may exist and should be considered, particularly when an increasing number of our population are currently overweight or obese.
